# 

**Published:** 1829-02

**Authors:** 


### NECROLOGY.

**DIED,** near Lexington, Ky. in the month of December last, George J. Ratrie, M. D. Dr. Ratrie fell a victim to pulmonary consumption, at the age of 30. He had received a regular medical education, but made it subservient to the practice of Dentistry; which he pursued with skill and assiduity, in many towns and cities of the west and south-west. In this important but neglected branch of the profession, he was really a useful man; and possessing sound principles, united with amiable manners, the people among whom, at various times he carried the benefits of his skill, could not have failed to regret his death.

—— In this city, in the month of February, Doctor Trueman Bishop, Physician and Minister in the Methodist Episcopal Church. Dr. Bishop was a man of gentle manners, exemplary piety, and impressive, unaffected, pulpit eloquence. He had not practised physic to much extent for several years; but at the time of his death, was vice president of the First District Medical Society of Ohio, and a member of its Board of Censors.

—— At Newport, Ky. in September last, Dr. Thomas Hinde, at the advanced age of 92 years.

—— At New Haven, Connecticut, within the present month, Nathan Smith, M. D. one of the professors in the Medical Department of Yale College.

Our next number will contain biographical sketches of both the last named gentlemen.

